# Implementation of Wayfinding Signage in Public Hospitals and Its Evaluation Towards Quality Improvement

**DOI:** 10.7759/cureus.65435

**Published:** 2024-07-26

**Authors:** Biswajeevan Sahoo, Jawahar S K Pillai, Sameer Md, Mukunda C Sahoo

**Affiliations:** 1 Hospital Administration, All India Institute of Medical Sciences, Bhubaneswar, Bhubaneswar, IND

**Keywords:** information education and communication (iec), fire safety signage, quality improvement and patient safety, signage, hospital wayfinding, total quality management

## Abstract

Background*:* Signage is important in any public place. It involves information about nomenclature, wayfinding, service timing, emergency preparedness, and regulatory compliance. It plays an important role in helping the patient reach their desired destination with minimum difficulty, thereby enhancing the patient experience. The hospital under study is a vast facility encompassing 130 acres. Outpatient services have a monthly footfall of nearly 1 lakh patients, and there are about 1,800 admissions per month. Patients and visitors are usually unaware of the facility and face difficulties in wayfinding amidst the large number of patients. Due to difficulty in wayfinding, the patients often seek the help of hospital staff to reach the desired locations. Trilingual signage (English, Hindi, and regional language - Odia) was installed in a 960-bed tertiary care public hospital in Eastern India as a Quality Improvement initiative towards the goal of a patient-friendly hospital.

Objective: The study aims to evaluate the multi-lingual signage system in the hospital and recommend suggestions.

Methodology: Wayfinding or the signage work was undertaken as a Total Quality Management (TQM) Project in the study setting, conducted over 36 months. The effectiveness of the signage system was evaluated using a questionnaire-based survey among the patients, attendants, and other visitors.

Results: Color-coded multilingual signage installed in the hospital block and its surrounding areas included the naming of various areas, way-finding, and safety signage. The most difficult areas to find were ICUs (35.6%), OT (31.1%), and laboratories (31.1%). Additionally, 98% of the participants could reach their desired destination but had to double-check with the staff.

Conclusion: It was evident from the study that hospital signage plays a crucial role in hospitals by improving wayfinding, enhancing the patient experience, and promoting safety and emergency preparedness. Signage also indirectly contributes to better patient care. In large teaching hospitals, human assistance is also necessary for wayfinding.

## Introduction

A signage system is a visual design that helps in identification, guidance, explanation, warning, and other functions via a combination of text, graphics, and colors. Signage is a comprehensive term that primarily includes names and way-finding. The term "wayfinding" was first coined by Kevin Lynch [1] in year 1960 and refers to the human ability to reach a destination in unfamiliar as well as familiar environments [2].

Hospitals are complex places where patients often face difficulty in navigating the facility. Hospitals in the study setting is a new institution established in the year 2012. It is a large institution and has several multi-storied buildings with numerous departments, wings, or sections. Navigating through such a complex layout can be confusing for the patients and visitors who are usually unfamiliar with the facility.

Patients who visit hospitals are usually in pain and stress due to their underlying medical condition. Their emotional state can affect their ability to navigate through hospitals, and, on the contrary, complex facilities can add to their stress level. They may also have physical limitations such as visual impairment or limitations in reading and understanding the hospital signage. Further, in times of emergency or urgency, the quick location of desired areas is crucial. Given these complexities, hospital signage is important and should be multilingual and have universally recognizable symbols.

Rationale

The study setting is a 960-bed tertiary care teaching hospital located in eastern India. It is visited by about 1 lakh persons per month in the outpatient units, which include patients, their attendants, and visitors. It has eight floors having 32 outpatient departments, 37 wards, 17 critical care units, 25 modular operation theatres, a maternity care complex, a dialysis center, a basement, a trauma and emergency unit, 12 departments for support services, and about 70 counters for registration, billing, sample collection, report dispatch centers, medicine dispensing, etc. The approximate floor area of the hospital building is 1.2 lakh square feet. In such large buildings, well-designed signage is essential for patient care and safety.

Regular complaints and feedback were received from the patients and staff in the administrative control room about the lack of correct and adequate signage in the hospital block. This resulted in a waste of valuable time for the healthcare workers, as well as the patients and therefore delayed the process of patient care. In addition, to the above, hospital signage is a mandatory requirement for the safety of the staff and patients under the Kayakalp guidelines by the Ministry of Health and Family Welfare, Govt. of India.

The objective of the study included the implementation of wayfinding signage in public hospitals and the evaluation of its effectiveness.

## Materials and methods

Wayfinding or the signage work was undertaken as a Total Quality Management (TQM) Project in the study setting, conducted over a period of 36 months. The TQM [3] is a proven quality management approach for long-term success through customer (patient) satisfaction [4,5]. It is evident from studies that the TQM strategy has a strong positive effect on quality performance and patient satisfaction and thus can influence the sustainability of the activities [6,7].

**Figure 1:**
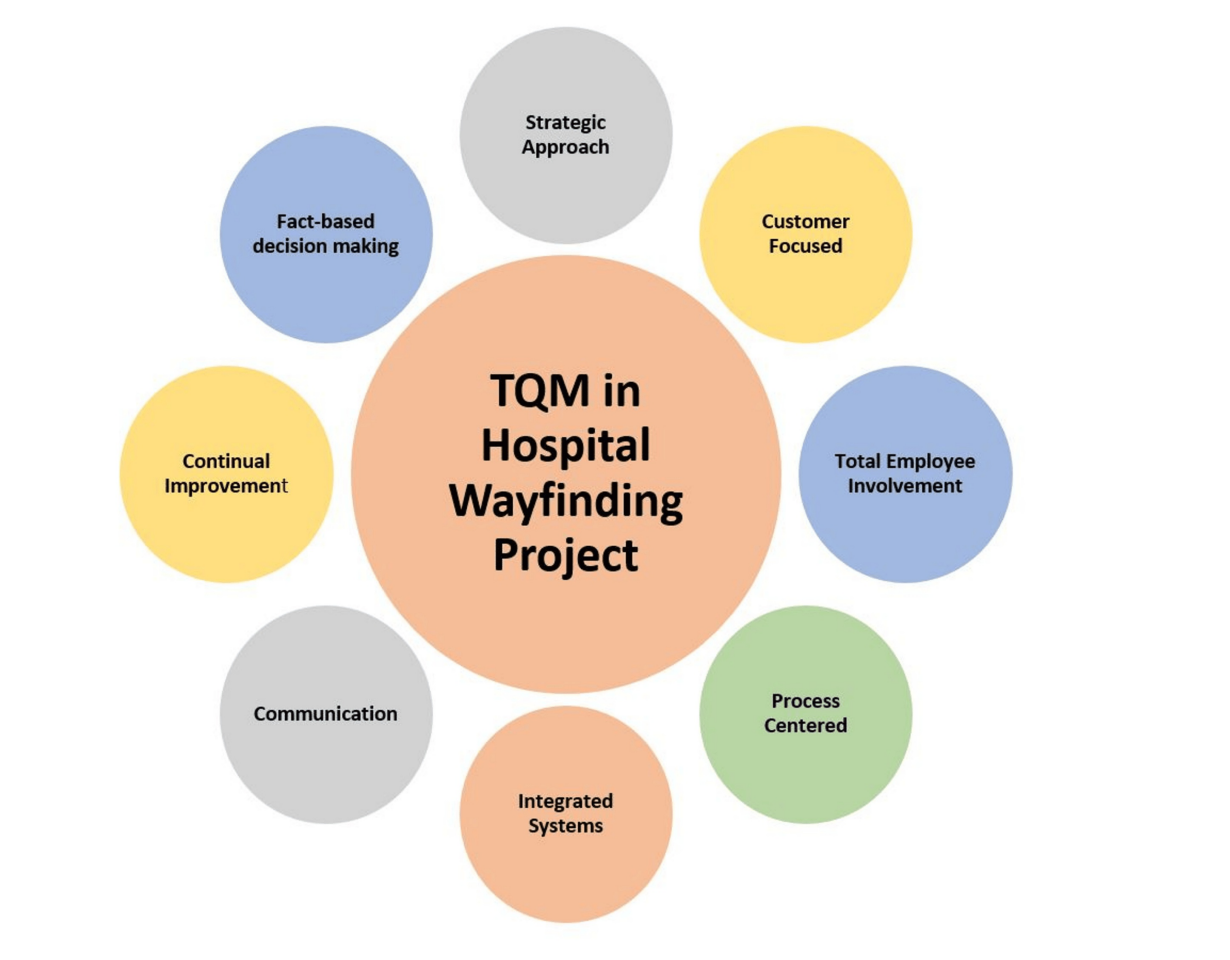
TQM Model of Quality Improvement TQM: Total Quality Management

The project started in the year 2018. Being a young institute, various surgical and medical specialties and super specialty services were gradually getting operationalized. Individual departments were using temporary signage such as paper printouts and stickers for the convenience of staff and patients. Being a large hospital, wayfinding was a challenge for both patients as well as staff. The majority of existing signage was in English and was not uniform. Thus, standardization of the signage system was necessary as it affected the patient care services in many ways. The TQM approach (Figure 1) focuses on customers (in this case the patients), employee involvement, process-centric, integration of systems, systematic approach, continual improvement, fact-based decision-making, and communications and therefore was the suitable model for the successful execution of this large-scale project.

The activities under each element of the TQM are mentioned below.

Strategy and systematic approach: To begin with, the Hospital Signage Committee was composed of faculty members from different departments such as hospital administration, community medicine, nursing, engineering, finance, purchase, and others. A road map was prepared by the committee for the development of wayfinding systems in the hospitals. National Building Code [8-10], Draft Indian Standards Safety Colours, Safety Signs and Accident Prevention Tags by Bureau of Indian Standards [11], National Disaster Management Authority (NMDA) Advisory for Hospitals [12], NABH Accreditation Standards for Hospitals [13], and Kayakalp Guidelines [14] by the Ministry of Health and Family Welfare (MoHFW), Govt. of India, were reviewed along with available scientific literature. Hospital wayfinding was broadly categorized into internal and external signage, which was further classified into nomenclature, number, service timings, directional or wayfindings, general information and instructions, safety or precautionary signage, educational (information education and communication - IEC), process signage, contact information, statutory signage, and emergency signage. Considering the type of installations as per their location, the signages were also categorized as wall-mounted, hanging, and projections.

**Figure 2:**
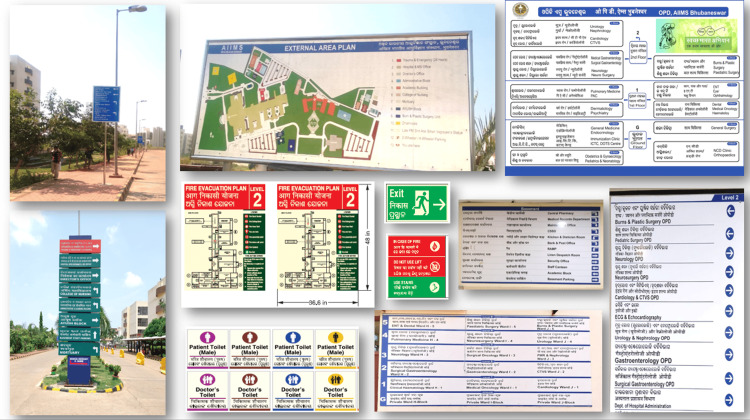
Outdoor and Indoor Signage

An external consultant was engaged through quotation in the initial period of the study to collect the baseline information about the hospital wayfinding requirement, the specifications for signage materials as per standards, the feasibility of installation in respective areas, and the financial estimation for the completion of the work. Subsequently, an agency was selected through the tender process for the actual execution of the work. A comprehensive list of items was prepared for the hospital block and installed (Figure 2).

Total employee involvement: Under the supervision of the Signage Committee, a ground team was constituted with resident doctors of hospital administration, junior engineers, nursing in-charges, fire safety personnel, sanitary supervisors, and members of the selected agency engaged in signage work. The team conducted the site survey through direct observations as per the list of signage items. The site survey was an internal survey conducted to verify the need for signage in that area and the feasibility of its installation. They also conducted informal interviews with the staff to understand their needs and to check the appropriateness of the signage in their respective areas. Based on their suggestion, the content and location of signage were revised. A timeline was followed as per the tender document for each phase of work.

Integrated systems: Digital television sets installed in the outpatient units were digitally integrated for multiple displays of floor directories, outpatient department (OPD) schedules, IECs, and other types of common information. The static signage and patient education videos were played on the TV network. Integration of wayfinding systems with the Hospital Management Information Systems (HMIS) was proposed with the support of the IT division and the electronic and telecommunication section of the hospital engineering division. It was also proposed for digital navigation systems with present-day mobile technology such as Alexa, Google Assistant, Apple Siri, and other similar platforms supported with artificial intelligence subject to infrastructural and financial feasibility at the institute.

Patient-focused: Considering the geographical location of the institution, all signage contents were prepared in three languages, namely, Odia (regional), Hindi (National), and English (Official). The agency set up a workstation with a content writer and designer. The institution's Language Cell (Raj Bhasha Cell) was also consulted. The draft contents prepared by the agency were verified by the ground team with the specifications, location, color contrast, font size, and appearance. The physical location of each signage board was pilot-tested with visitors or patient attendants at the actual sites with attention to the height of the installation site and font size in the actual print (3 inches for upper case letters and 2 inches for lower case letters), as well as visibility from a distance ranging from 10 feet to 50 feet. Pictographic content was emphasized as compared to the text for quick and easy interpretation. Floor directories and wayfinding in front of lifts and corridors were tested for correctness based on real-time feedback from the patients. Several medical terminologies mentioned in the signage did not have exact translations in the regional language for which the English terminology was written in the regional language to have a phonetic resemblance to prevent the patients being misguided within the buildings. The color coding system for individual blocks was incorporated for easy identification of the locations by the patients.

Fact-based decision-making: The observation of the ground team helped review the content of the signage with the staff of respective units. The quality of printing and fixing materials was verified with material testing reports from authorized laboratories. The decisions for the selection of materials, alphabet font size, content, and locations, were fact-based concerning the feedback, reports, and physical survey findings.

The signage committee recommended the signage concerning the standards for final printing and execution. Approximately 5,000 signage boards were installed. The fire signage was reviewed by the Fire Safety Team and the Engineering Team. The installation was completed in a period of six months. The recommendations of the Hospital Infection Control Team, Help Desk Team, and Registration Counter Team were considered for the development of the content of the IEC materials.

Continual improvement: Towards the end of the year 2019, the hospital underwent major changes in terms of opening new wards, new modular operation theatres, intensive care units (ICUs), and other super-specialty units. Similarly, in the year 2020, there were several modifications in the hospital setting because of the COVID-19 pandemic. Additional signages were included as per the government’s direction because of the pandemic.

As a continuous quality improvement, periodic meetings were conducted with all stakeholders, and inputs were collected from heads and staff from individual units or departments and patients. Some of the changes were incorporated into the existing designs, and those beyond the scope of the project were rejected. This measure was taken to prevent the overcrowding of signages in a particular area. In the process of verification, signages were rejected for spelling errors, pictographs not corresponding to the text, and incorrect color coding.

Process-centric approach: The process of the signage project was defined and implemented as per government rules. The step-by-step process was developed for the whole project and was executed through the tendering systems by entrusting the responsibility of monitoring to the Signage Committee for the timely completion of the project. Duplication of work activities was minimized through teamwork.

**Figure 3:**
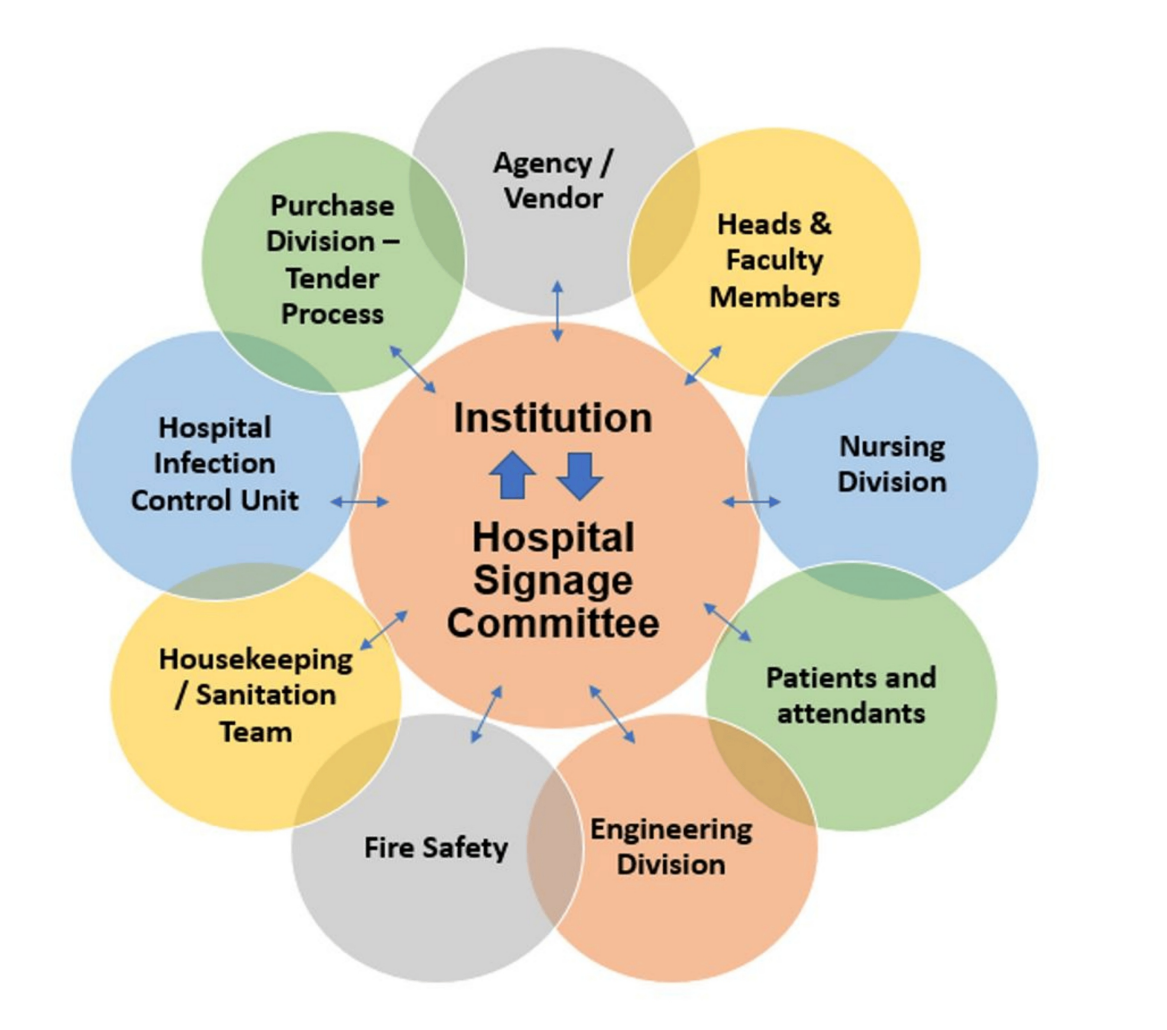
Communication Process in the Signage TQM Project

Communication: The TQM approach emphasized the interdepartmental communication between the signage committee the senior management of the institution and all the stakeholders (Figure 3).

**Figure 4:**
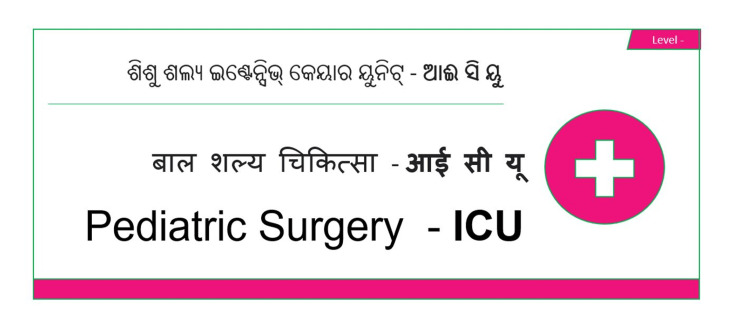
Trilingual Signage

The other aspect is the communication of the information through the signage system to the stakeholders. All signage had three languages. Considering the design of the hospital building, each block was assigned a color and an alphanumeric code. The Outpatient department was marked deep blue, critical care areas were marked pink, operation theatres were marked green, and wards being multiple blocks were marked other colors. The walls are marked with the same color stripe for easy identification of the area (e.g., ‘H+2 ward’ indicates the H-block second-floor ward). The text was always associated with a symbol and floor level (Figure 4).

All these internal signages were printed on vinyl sheets and pasted on acrylic boards. The thickness of the acrylic boards was 6 mm and was permanently fixed to the wall using screws. The hanging signage and projection signage were installed using D-clamp steel chains and aluminum channels, respectively. Fire safety signage boards used auto-glow vinyl print materials on acrylic boards. This included fire safety signage - rescue, alarm, confine, extinguish (RACE) and pull, aim, squeeze, and sweep (PASS), fire emergency contact numbers, fire evacuation maps, assembly areas, and directional arrow markings for fire exits. IECs were installed in common areas like waiting rooms, corridors, staircases, entry and exits, public toilets, etc. These included general IECs such as patient rights and responsibilities, disposal of wastes, hand hygiene, and infection prevention and control practices. The electrical rooms and the air-handling units (AHU) rooms were numbered as per the directory of the engineering team. Combined pictographic signage with room numbers and safety symbols are installed to prevent unauthorized access. Emergency contact number signage included information on the administrative control room, fire safety, security, medical gas supply, and engineering (electrical, plumbing, lift, and air conditioning) maintenance department.

External signage of pylon-type totems and pole-mounted signage included information on institution maps and wayfinding for various blocks and buildings. Both day and night viewing was ensured with LED backlighting in the pylon-type totems and the retro-reflective sheets in the pole signages ensured night visibility. The font size of a minimum of three inches and a height of 72-78 inches from the floor level was maintained for easy visibility. These were fixed permanently to the ground with reinforced cement concrete. Other external signage included the signage installed in the basement, internal roads, public parking, and outer areas around the hospital block within the campus.

Evaluation: A cross-sectional survey was conducted among the patients, their attendants, and visitors using a questionnaire that was developed in consultation with domain experts. A pilot study was conducted for validation of the questionnaire. A convenient sampling technique was used in the survey. Healthcare staff were excluded from the survey as they were assumed to be oriented to their workplaces. The findings were analyzed statistically and inferences were drawn.

## Results

The TQM methodology resulted in the development of robust color-coded, multi-lingual signage in the hospital block. Signages were installed in an approximate total area of about 1 lakh square feet.

About 5,000 units of signage boards were installed in the OPDs, wards, ICUs, trauma and emergency departments, operation theatres, and other areas.

Standardization of the signage contributed to the improvement of accessibility of various areas in the hospital block. Fire and other safety signage helped in statutory compliance. The signage system significantly improved the aesthetics of the hospital, and as a result, the institution was awarded the prestigious Kayakalp National Award (first prize - category B institutions) in the year 2019-20 and 2021-22 by the MoHFW for promoting cleanliness and enhancing the quality of healthcare facilities in India.

Findings of the survey: A total of 507 persons participated in the survey (Table 1). 

**Table 1:** Details of the Participants

Details of participants (n=507) in the survey		
Category	Particulars	Percentage of Participants
Category of participants	Patients	48%
	Patient attendants	43%
	Visitors	9%
Native language	Odia (regional language)	50%
	Bengali	36%
	Hindi	11%
	Others	3%
Literacy status	Yes	100%
	No	0%
Visual impairment		Nil
Any other impairments		Nil
Frequency of visits to the hospital	Visited the hospital 2-3 times in the last 1 year	35.60%
	Visited the hospital 2-3 times a month	26.70%
	Visited the hospital once in the last 1 year	20%
	Visited the hospital once in the last month.	17.80%

**Figure 5:**
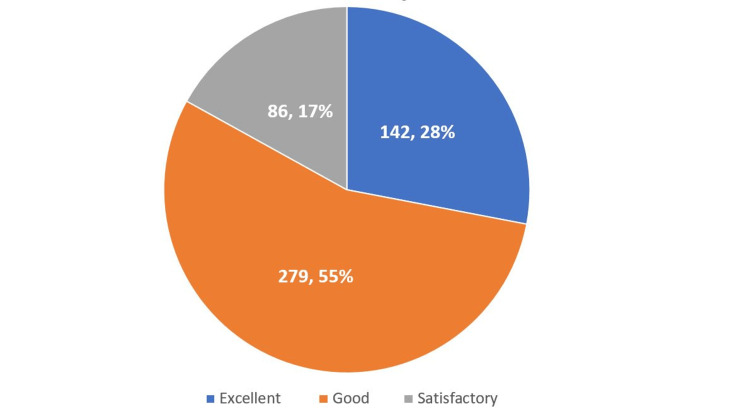
Overall Patient Satisfaction With the Hospital Wayfinding Signage System

Concerning the overall rating of the signage system, 55% of the participants rated it as good, 28% of participants rated it as excellent, and 17% rated it as satisfactory (Figure 5).

Among the respondents, 98% mentioned that they had to reconfirm the destination inside the hospital with the staff or other patients though the signage said it was their desired destination. About 91% of the participants were not able to reach their destination only following the signage without the help of others. Additionally, 84.4% of participants mentioned that they kept asking about the location, while they were on their way. Participants expressed that they had to cross-check with the staff to ensure the right wayfinding. Moreover, 91% mentioned that they lost their way initially but managed to reach their destination, and 31% of participants lost themselves in the hospital (Table 2). 

**Table 2:** Participants' Responses on Reaching the Desired Locations

Questions	Did you find or reach the desired location using the signage?	Yes (%)	No (%)
Response - 1	I reached the desired location without getting help from anyone.	6.67	91.11
Response - 2	I reached the desired location but had to reconfirm with the staff/ other patients	97.78	2.22
Response - 3	I kept asking staff/ patients while looking for the desired area	84.44	13.33
Response - 4	I was lost initially but still managed to reach the location	91.11	6.67
Response - 5	I was lost and had to call others to guide me	31.11	57.78

**Figure 6:**
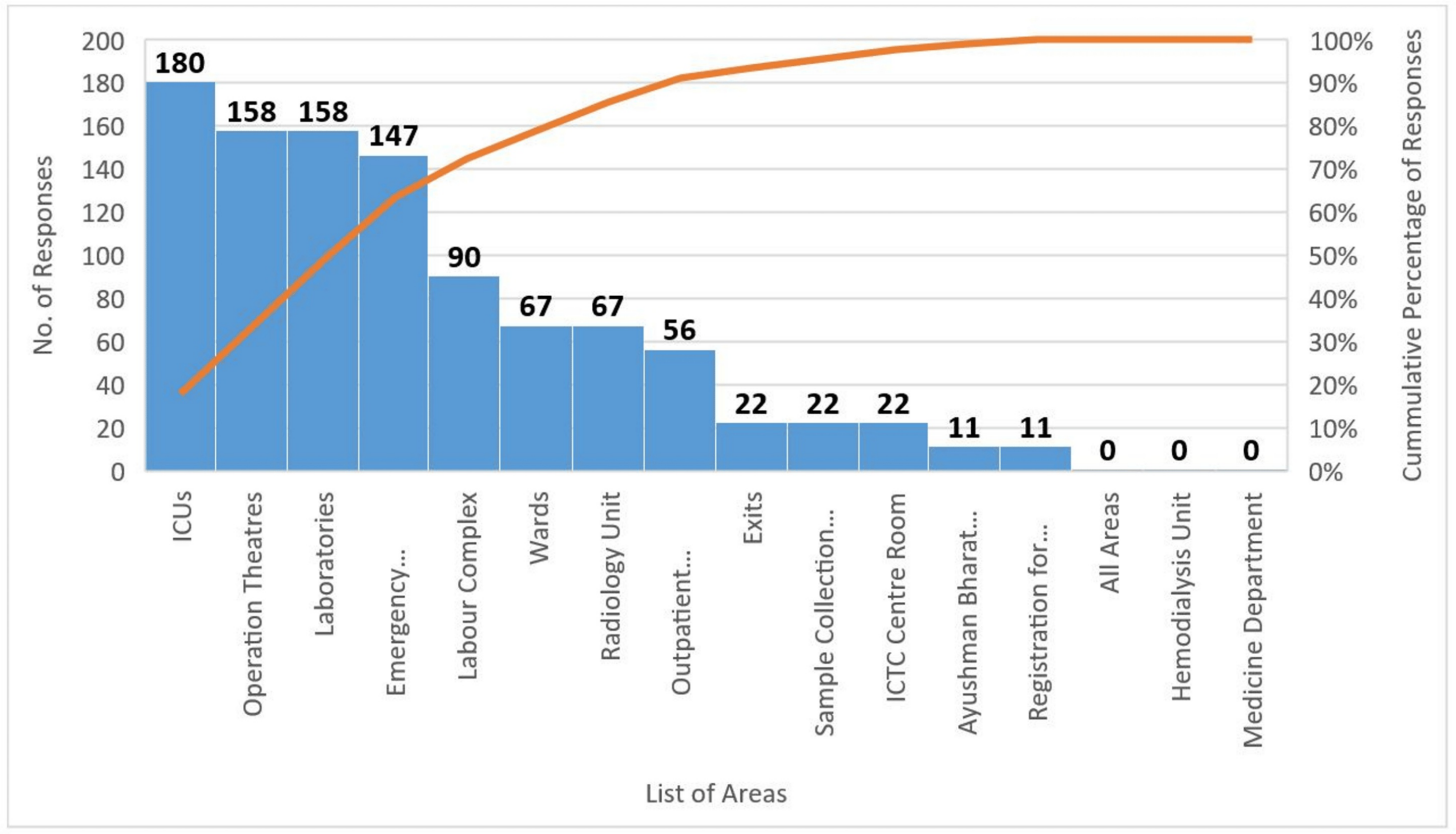
Pareto Chart of Difficult-to-Reach Areas in the Hospital

The difficult-to-reach areas in the hospital were also identified using the Pareto analysis (Figure 6). Specifically, 35.6% of participants found it difficult to reach ICUs, followed by the operation theatres (OT) (31%), laboratories (31%), and the emergency department (28.9%).

**Figure 7:**
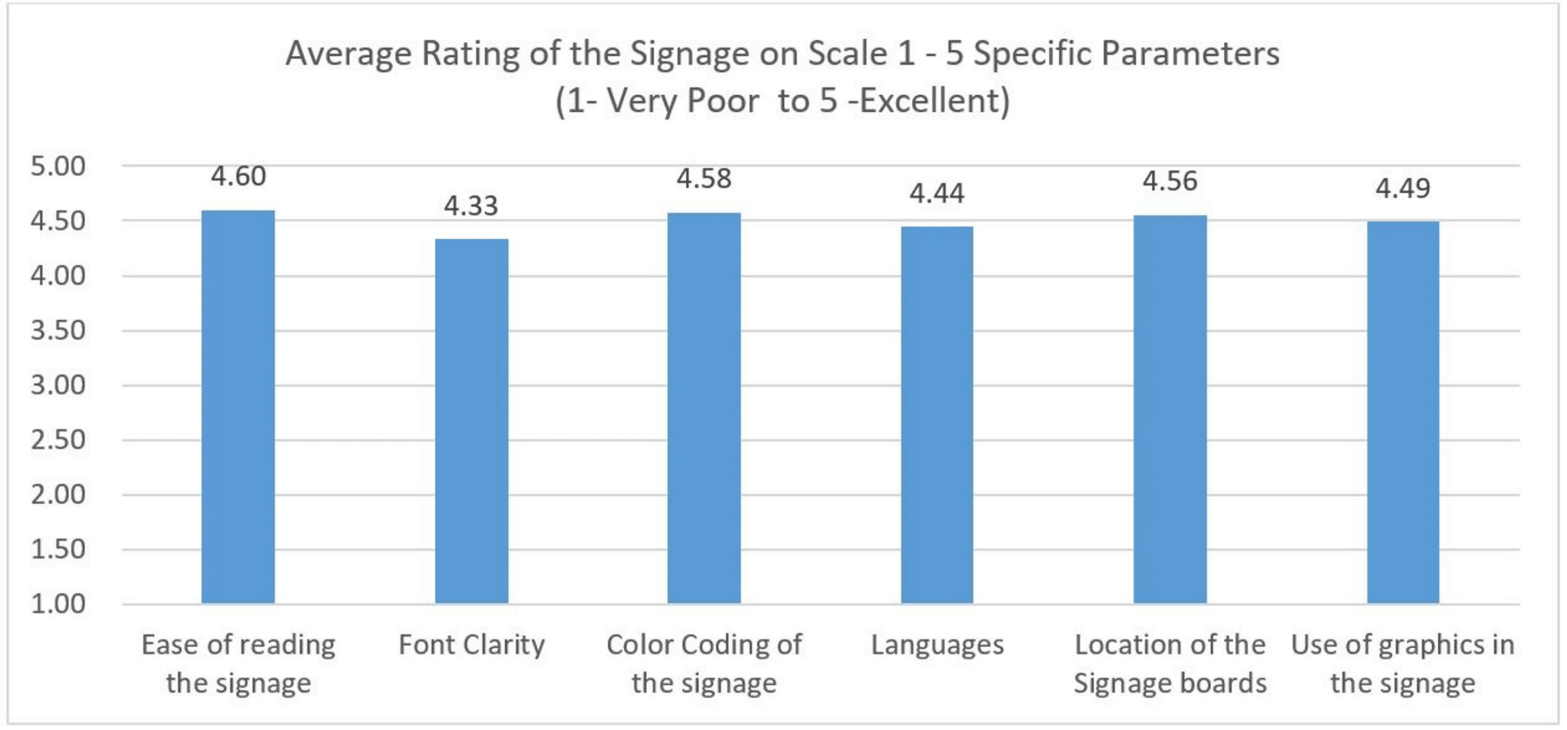
Rating of Quality of Signage - Likert Scale Rating

The quality of the signage (Figure 7) was scaled using the Likert scale (1 - Very Poor to 5 - Excellent), and the mean score for ‘ease of reading’ the signage was 4.6, followed by color coding (4.58), location of the signage board (4.56), use of infographics in the signage (4.49), use of language (4.44), and font clarity (4.33).

**Figure 8:**
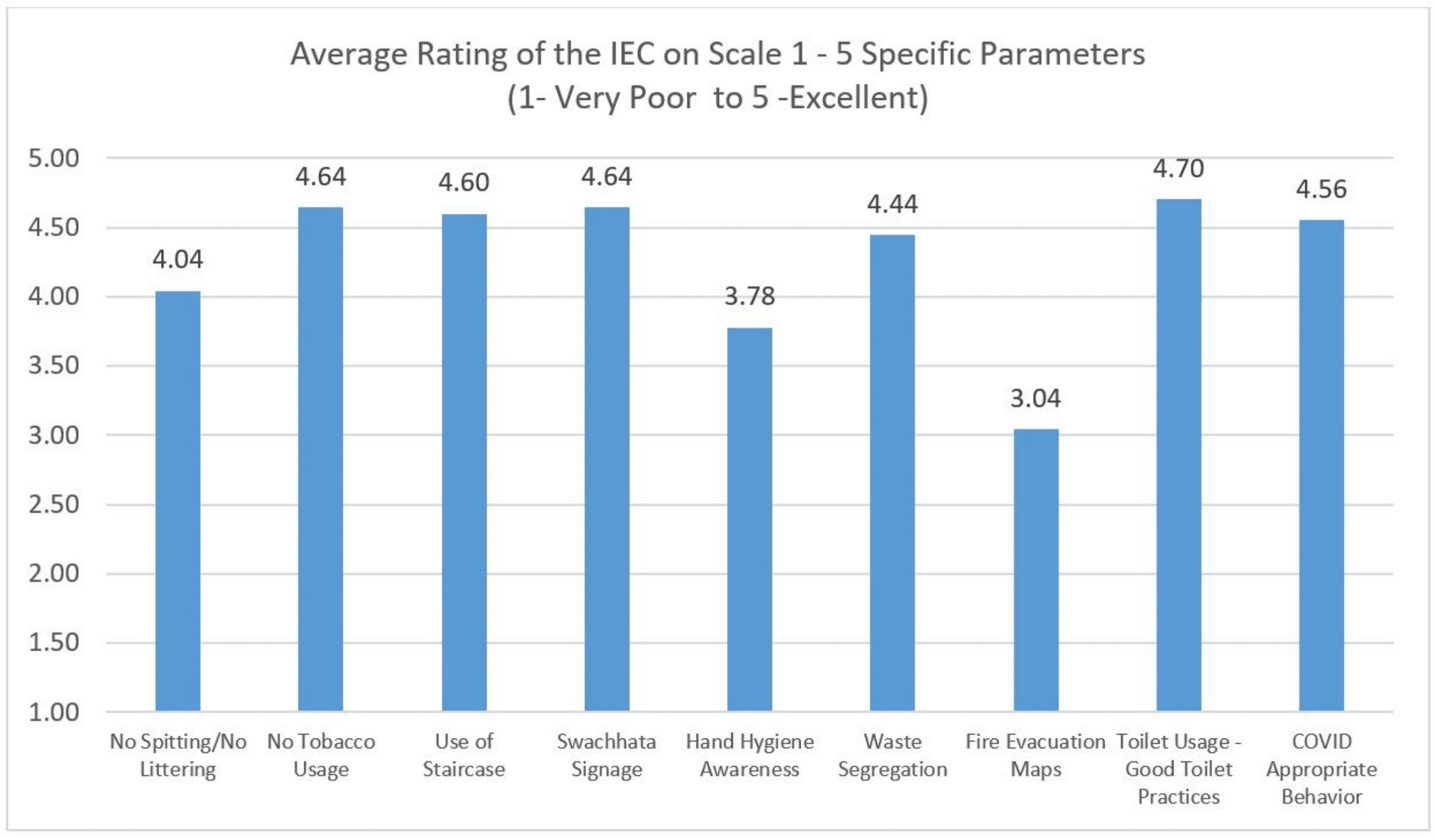
Rating of IEC - Likert Scale Rating

The IEC signage was also scaled using the Likert (Figure 8). The mean score for the IEC - Toilet Practices was highest, followed by Swachhata (meaning cleanliness) (4.64). The hand hygiene IEC and Fire Evacuation IEC have the lowest with scores of 3.78 and 3.04, respectively.

The data for the quality of signage and the rating of IECs was analyzed at a 95% confidence interval, and the level of significance was <0.05 for both categories.

**Figure 9:**
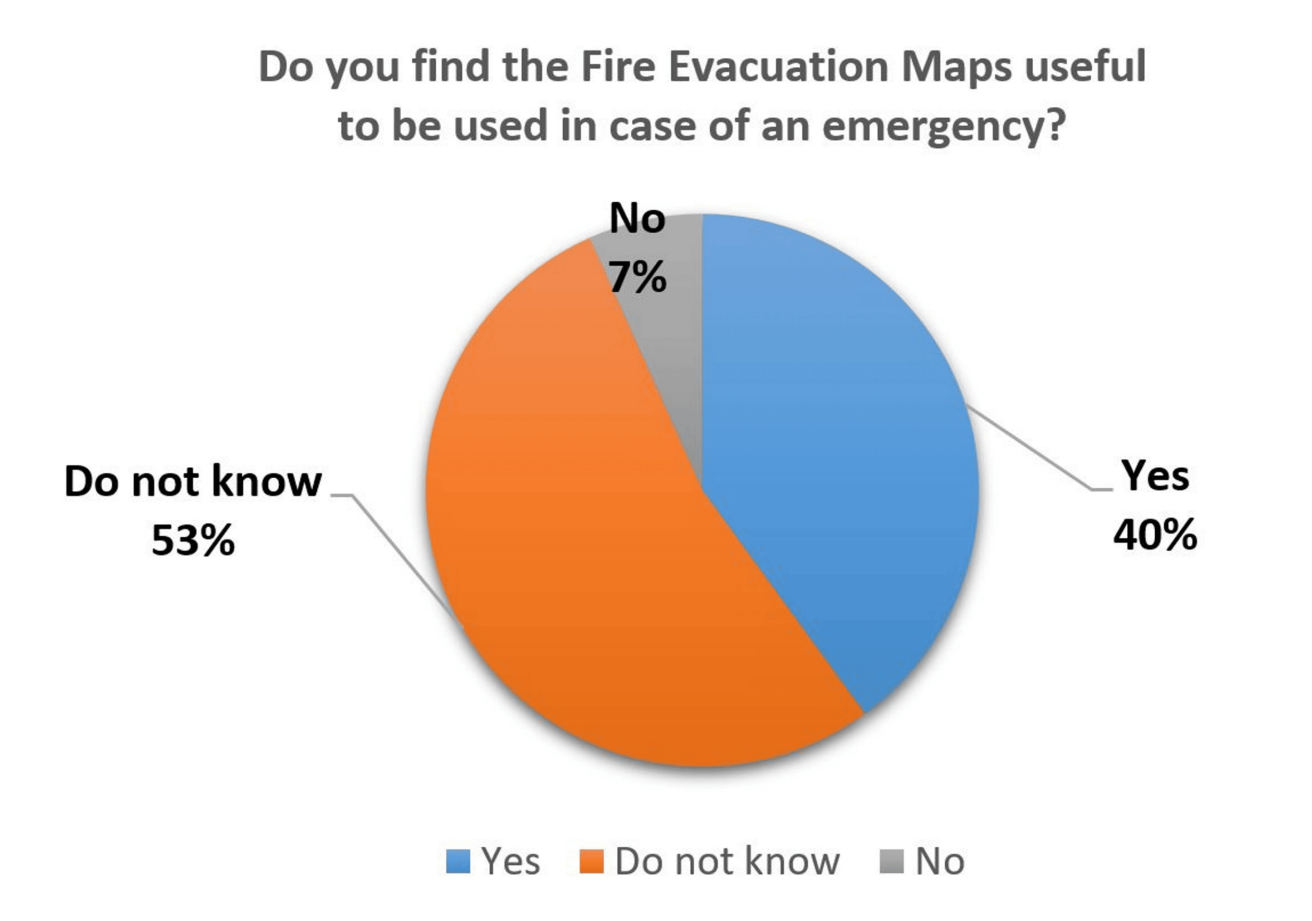
Fire Evacuation Maps - Usefulness for Patients

**Figure 10:**
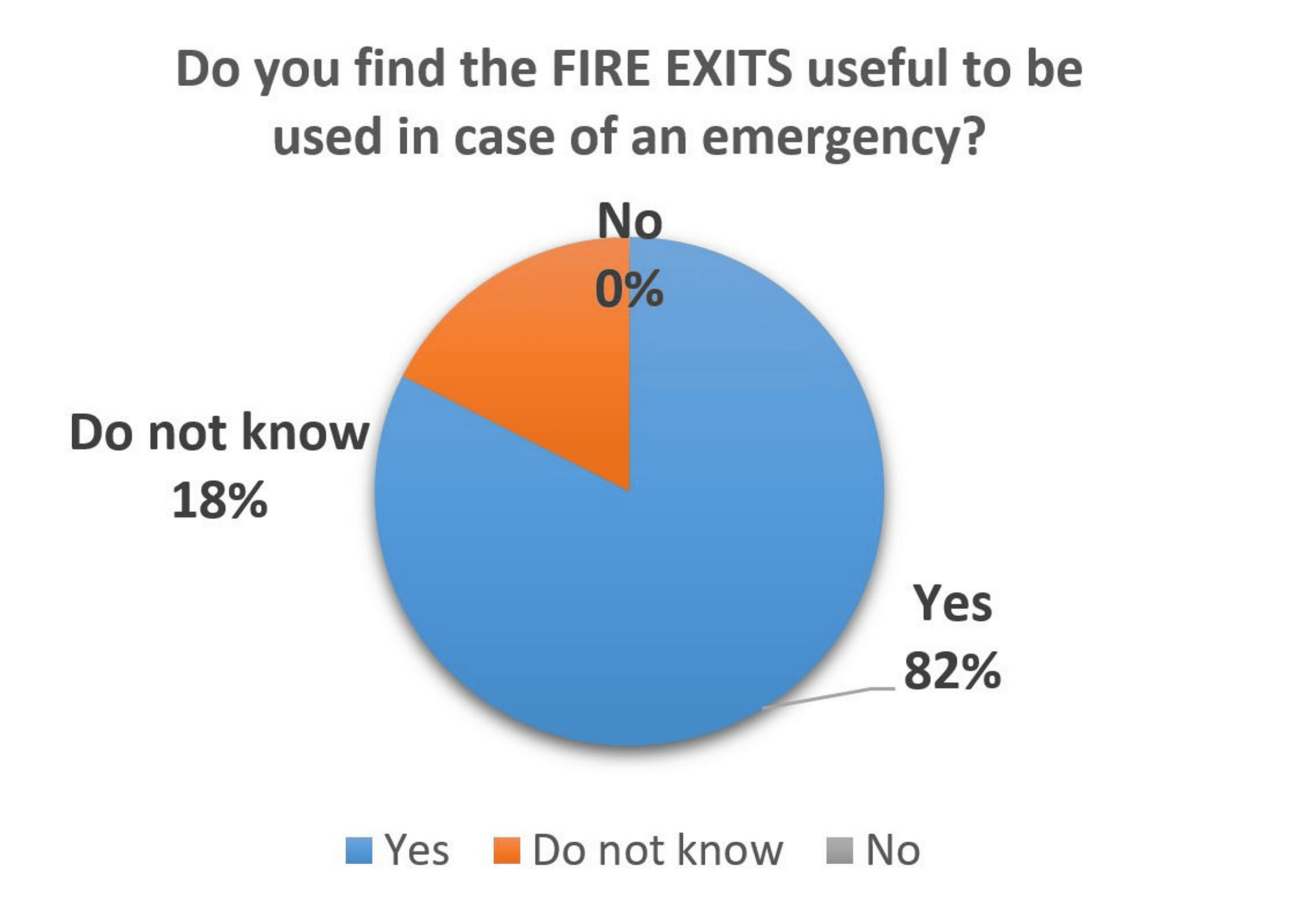
Usefulness of Fire Exit Signages Among Patients

Concerning the fire signage, a significant number of participants (53%) did not know about the fire evacuation maps (Figure 9), whereas 82% found the fire exit signage to be useful and can be used during an emergency (Figure 10). There is a lack of awareness about the fire safety signage and evacuation maps among the people visiting the hospital.

Several important points were identified through open-ended questions. Overcrowding and the long queues make it difficult to see the name board and identify the location correctly. Illiterate patients do not know about the common symbols or pictographic signage and therefore seek human assistance. A few of the participants opined that the waste segregation signage in the common areas is insufficient and should be prominently displayed. Color bands in the individual blocks confused the patients as they were not informed about it at any point of time during their stay in the hospital. Participants mentioned that it is easier to ask someone for directions than to look for the wayfinding inside the hospital.

All the participants opined that the signage has significantly reduced the time spent accessing the areas and services. The need for digital signage was mentioned by 84% of participants. Additionally, 33% of participants opined for better locations of the signage, and 13% suggested for bigger signage.

## Discussion

Installation of hospital signage in large tertiary care hospitals is a herculean task (Table 3), and it could be successfully developed and efficiently executed using a strategic approach of TQM.

**Figure 11:**
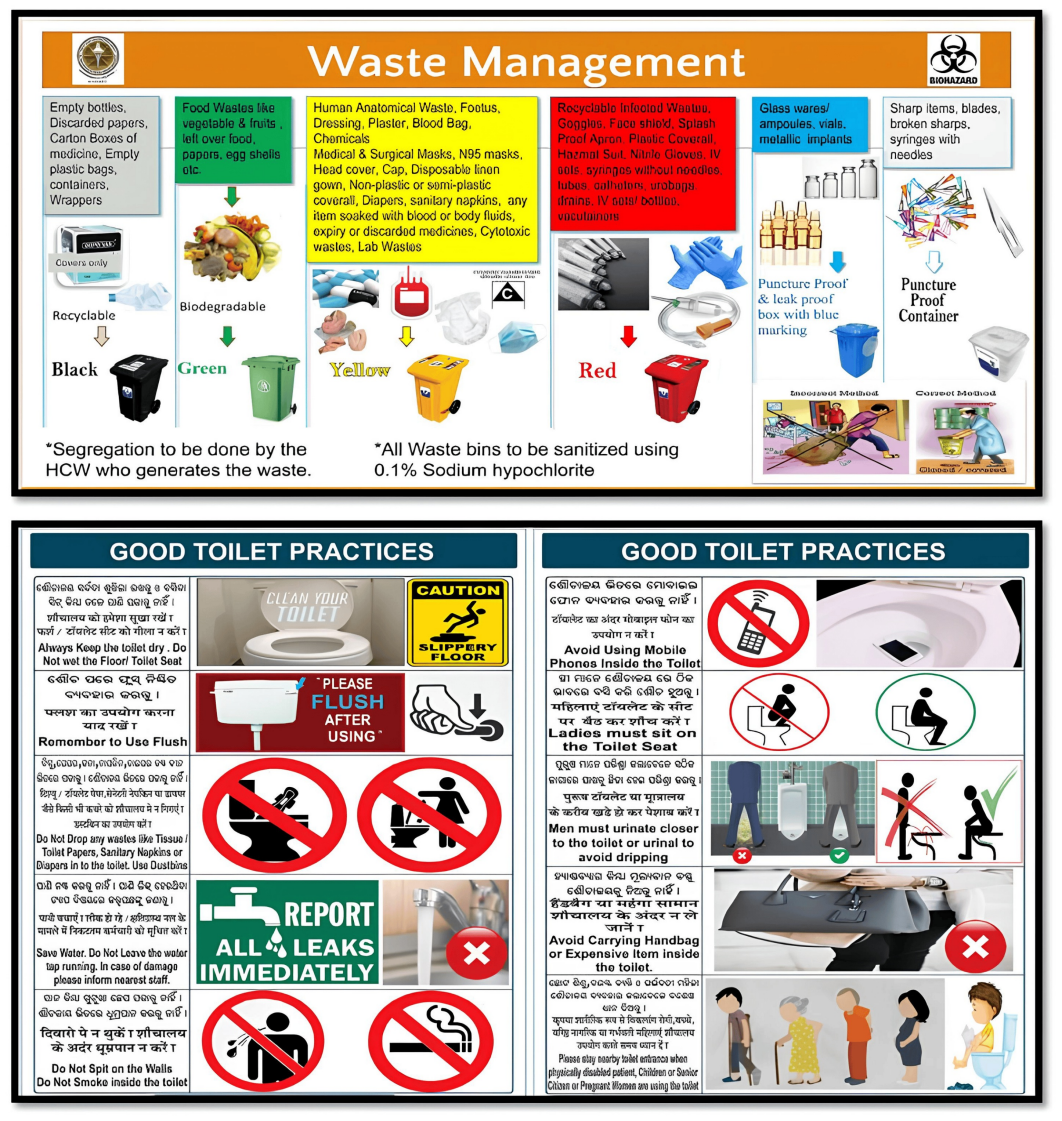
Information, Education, and Communication (IEC)

The wayfinding system is composed of four types of signs: directional, informational, identification, and regulatory. It provided information on the names, locations, timings, and wayfinding and also proved to be an effective mode of educating patients visiting the hospital through the IECs (Figure 11). It is not restricted to signs, but also uses architecture, colors, lighting, or technologies. This was evident from the study. Similarly, it is not only restricted to the clinical areas but also the non-clinical areas such as electrical rooms and support services, to prevent potential hazards and ensure safety.

Signage improved hospital appearances and helped to build trust among patients and attendants. It also contributed to the Kayakalp National Award for the institute.

Floor directories and wayfinding signage helped to reduce the dependency of patients on the helpdesk and security personnel for inquiries about the departments and processes. Entry of unauthorized persons in vital areas such as operation theatres, critical care areas, medical records, and electrical panel rooms was prevented through proper signage. Numbering systems of consultation rooms, beds, electrical rooms, HVAC (heating, ventilation, and air conditioning) rooms, toilets, staircases, lifts, and other areas helped the patients and staff for quick identification. It also helped in the maintenance of the areas for patient convenience. Directional signage helped streamline internal traffic, thereby preventing congestion.

Display of emergency contact numbers such as fire safety, security, lift, maintenance, and help desk in all areas provided means for essential communication. Contact details for feedback and suggestions installed in all areas helped to build the confidence of patients in the system by raising their concerns, thus making the hospital patient-friendly.

Statutory safety symbols are also installed in departments of radiodiagnosis, pharmacy, chemotherapy units, and waste collection points, showing the commitment of the institution to patient safety. The hospital also has floor directories reducing the burden on the helpdesk team.

The quality of the signage on the parameters of ease of reading, location, language, pictographic representation, and font clarity was rated high. It is interesting to note that, though the majority of the participants were satisfied with the signage system, still they took help from staff and other patients to reach their destinations. This is a common challenge in large hospitals and patients feel that it is necessary to be sure about the location to save time and energy.

Rodrigues et al. [15] in their study on designing effective healthcare signage systems mentioned the problems due to poorly designed signage. This is attributed to the lack of awareness of the guidelines by the developers. They opined that there is a need for uniform signage system guidelines that can help build and implement effective and efficient signage systems. These guidelines should include language and terminology, text formatting, symbols and pictures, colors, placement, dimensions, illumination, visibility, and legibility, and the inclusivity characteristics should be specific to the cultural contexts. Similar results were also observed in the present study, and uniformity was noted in all signage.

Rousek et al. [16] analyzed the standardized healthcare pictograms and the effects of color contrasts and complexity. The Participants included normal and visually impaired persons. They concluded that certain color contrasts can hinder or help comprehension of the signage. They recommended that the standardization of signage in the hospital can aid in the cognitive thought process of detecting and interpreting the signage. Though this factor of color contrast has been considered by the hospital administration, there is no system for visually impaired patients in the study setting.

Lee et al. [17] conducted a literature search for the patients’ wayfinding experiences in the improvement of quality of care. They concluded that a good signage system reduces nursing idle time and is a cost-saving strategy for hospitals. A similar conclusion can be deduced from the present study. If the patients are well-guided with the help of signage, it directly benefits the staff in terms of time saved in guiding individual patients.

Apelt et al. [18] mentioned that pictograms communicate better among populations with high illiteracy rates, whereas concrete symbols are better understood than abstract symbols.

Morag et al. [19] mentioned the relationships between architectural features and wayfinding experiences. These include layout configuration, visibility, lighting, and differentiation between the elements based on form, size, color, etc. In another study [20], the author developed a questionnaire to collect data about the subjective experiences of people with different abilities. The methodology was similar to the present study and uncovered the wayfinding problems in the hospitals. It emphasized that the signage should be based on the needs of the patient instead of the institute determining the parameters. However, regulatory and educational signages are to be as per local rules and institutional policies and the same was evident in the study.

Signage supports healthcare workers by saving time in providing routine information. Iftikhar et al. [21] studied the effectiveness of wayfinding in complex environments through desktop-based virtual reality experiments. They found that real-time environmental information and mobile information were influential in the orientation of the participants. This type of system can be adopted in a hospital setting based on feasibility.

MacKenzie et al. [22] focused on the need for a professional signage system in hospitals as the patients are generally unfamiliar and face trouble in finding their way inside large hospitals. A similar challenge was identified in this study where several patients felt lost in the large crowded public hospitals.

Leonard et al. [23] reviewed the existing signage in a children's hospital and developed improved signage that aims to convey essential information to parents, caregivers, and patients. The findings were similar to the present study and were noticeable, attractive, and easily understandable. Participative re-designing of the signage resulted in a sense of ownership/belongingness of the signage among the staff working in that area.

Mora et al. [24] surveyed wayfinding and a sense of orientation in the hospital among patients. The results were similar to the present study in terms of satisfaction with the signage system, and yet a majority of the patients asked for directions from the staff to navigate within the hospital.

Considering the technological advancement in the signage systems, the hospital should implement a digital signage system as it has the benefit of displaying multiple information in less space. It may appear to be costly, but it has many benefits such as ease of updating the information. Changes in the internal architectural designs or relocations of the departments are commonly seen in hospitals. It is difficult to make changes in permanent signages. On a similar note, institutions have moved into app-based mobile technology for assisting patients in large complex facilities such as hospitals. This can help patients with physical impairments navigate inside hospitals.

The rating of the fire safety signage was lower as compared to others. The participants lacked awareness of fire evacuation maps and often ignored them during their hospital visits. Thus, staff needs to educate the patients about fire safety during their hospital stay with minimum essential information.

In the study setting, patient navigators are available on each floor, which augment the signage system for providing information on wayfinding to the patients and others. This strategy has been adopted considering the number of patients visiting the hospital and also the large area of the hospital blocks. Therefore, human assistance is also needed.

Experiences of implementation of hospital signage could be useful to other institutions desirous of installing signage.

Hospital wayfinding and signage may change with time in terms of wear and tear or changes in the service provisions in the concerned areas. Therefore, periodic maintenance and review of the existing installations in the facility can be ensured by conducting facility rounds and taking necessary corrective measures.

The majority of the participants found the signage system to be helpful in wayfinding but also opined that existing signage was not sufficient for such a large hospital and needs upgradation through digitization.

Limitations

The study was conducted in a single hospital, and while most of the findings will be similar in other settings, there could be differences depending on the geographical areas, the content, the usage of language, directions, and the literacy of the public.

It did not cover the special signage requirement for patients with visual or physical impairments. The exclusion of staff in the questionnaire-based survey is also considered a limitation of the study. The digital signage could not be installed due to funding issues.

Understanding the signage is directly dependent on an individual's perception, literacy level, and state of mind in a hospital setting, thus limiting its assessment.

Recommendations

In order to ensure the sustainability of the quality improvement in the hospital signage system, an annual maintenance contract for the existing signage system, strict supervision, and monitoring are suggested. Exercises such as hazard identification and risk assessment (HIRA) and periodic facility rounds help to bring in long-term sustainability.

Since it is evident from the study that there is a lack of a digital signage system in the hospital, the use of technology for the improvement of the existing signage is strongly recommended. Recently, mobile technology such as Alexa’, ‘Google Assistant’, and ‘Siri’ in phones are widely used by everyone. Thus, AI-driven and mobile app-based navigation systems can be developed for hospitals.

Patient feedback is essential and should be considered in designing a good signage system that includes color contrast, visibility, readability, and interpretation of the graphics used. For the visually impaired, braille signage is also necessary. However, to overcome the challenge of a large number of patients in public hospitals, human assistance will always be needed as backup support.

## Conclusions

The trilingual signage system was developed using the TQM model for quality improvement. It was found to be impactful and showed high satisfaction among the patients, their attendants, and visitors. Large public hospitals are complex and need a standardized signage system to assist the patient navigate easily during his/her stay in the healthcare facility. The challenges, limitations, and scope of improvement were identified in the study and recommendations were suggested. The study can be used as a reference for the development or improvement of signage in other hospitals.

## Appendices

**Table 3:** Sample List of Signage

IEC Signage	List of Wards	Signage for Individual Wards	ICU Signages	OT Signages
Patient Rights and Responsibilities	Trauma Ward	Security Checkpoint	Surgical ICU	Wheelchair and Stretcher Bay
No Smoking, No Spitting. No Littering	Physical Medicine and Rehabilitation Ward	Name of the Ward	Surgical HDU	Name of OT Complex
Swachhata Campaign	Private Ward - H	Location of the Ward	Central ICU	Restricted Access Signage
Prohibition of Tobacco usage and other illegal substances	Private Ward- I	Ward Directory	Medicine ICU	Pre-operative Zone
Penalty imposition for open urination	Private Ward- J	Nurses' Station	Respiratory ICU	Post-operative Zone
Mercury Free Campus	Cardiology Ward	Store Room - Medicines & Consumables	Neurology ICU	Recovery Room
Single Use Plastic Free Campus	CTVS Ward	Store Room - Linen	Neurosurgery ICU	Operation Theatre No. _
Water Conservation	Nephrology Ward	Janitors Equipment Room	Hematology ICU	Hand Washing Area
Steps of Use of Hand Rub and Hand Washing	Urology Ward	Dirty Utility Room	Trauma ICU	Equipment Room/ Zone
5 Moment of Hand Hygiene	Burns and Plastic Surgery Ward	Soiled Linen room	Cardiology ICU	Clean Item Store / Clean Linen Room
Needle Stick Injury Protocol	Hematology Ward	Drinking Water	CTVS ICU	Sterile Item Store
Spill Management	Medical Oncology Ward	Pantry Room	Gastroenterology ICU	Medicine and Consumable Items Store /Area
Biomedical Waste Management	Neonatology and Obstetrics and Gynecology Ward	Biomedical Waste Collection Area	Surgical Gastroenterology ICU	Patient Counselling Room
Segregation of General Waste	Pediatric Ward	General Waste Collection Areas	Transplant ICU	Portable Oxygen Cylinder Area
5-S Methodology at the workplace	Obstetrics and Gynecology Ward	Bed Nos.	Pediatric ICU	Crash Cart Area
Fire Safety and Prevention	Psychiatry and Deaddiction Ward	Entry and Exit	Pediatric Surgery ICU	Faculty Room - Male / Female / Common
Hazardous Waste	Gastroenterology Ward	Procedure Rooms	Neonatal ICU	Nurse Room - Male/ Female/ Common
Cytotoxic Wastes	Surgical Gastroenterology Ward	Treatment Room	Signage for Individual ICUs/ HDUs	Residents Room
Radiation Hazard Caution	Surgical Oncology Ward	Isolation Room	Entry and Exit	Technician Room Male/ Female
Good Toilet Practices	Pediatric Surgery Ward	Duty Doctors Room	Visitor Timing	Staff Toilets -Male/ Female
COVID Appropriate Behaviour	General Medicine Ward - D	Faculty Room	Restricted Access	Toilets for Doctors - Male/ Female
Donning and Doffing Methods	General Medicine Ward - E	Duty Nurses Room	Pantry Room	OT Lounge
Patient Education Handbook on COVID-19	General Medicine Ward - F	Toilet Numbers	Store Room	TSSU Area
Use of Stairs	Orthopedics Ward - D	Seminar Room	Wheelchair and Stretcher Bay	Instrument Room
Fit India Movement	Orthopedics Ward - E	Nursing Incharge	Biomedical Waste Collection Area	Instrument Cleaning Zone
Unauthorized Access Restriction	ENT Ward	Faculty	Bed No.s	Instrument Packing Zone
Crowd Management	Endocrine and Metabolism Ward	Counselling Room/ Zone	Nursing Incharge	Biomedical Waste Collection Room / Area
Immunization Schedule	Radiation Oncology Ward	Patient Cloak Room	Nurses' Station	Dirty Linen Collection Room/ Area
	General Surgery Ward	Patient Toilets	Medicine Room	Changing Rooms - Staff - Male / Female
	Ophthalmology Ward	Staff Toilets	Patient Counselling Room or Area	Changing Rooms - Faculty - Male / Female
	Neurology Ward	Post-operative Patients Zone	Crash Cart	Changing Rooms - Residents- Male / Female
	Neurosurgery Ward	Dressing Room	Portable Oxygen Cylinders	Janitor Room - Housekeeping / Sanitation
		Crash Cart	IECs	OT Zoning Signage - Color Coding for Clean, Protective, Sterile and Dirty Zone
		Wheelchair and Stretcher Bay	Special Room for Medicine Preparation	OT Calling Bell Signage
				Emergency Exits
				Fire Signages
				Shoe Rack and Remove Your Shoes Signage
				Data Entry Section
				Security Checkpoint
